# Efficacy of seventh generation bonding agents as desensitizers in patients with dentin hypersensitivity: a randomized clinical trial

**DOI:** 10.1186/s12903-024-04352-0

**Published:** 2024-05-14

**Authors:** Sumaiya Shabbir, Shahbaz Ahmed, Syed Jaffar Abbas Zaidi, Sania Riaz, Huma Sarwar, Muhammad Taqi, Zia ur Rahman Khan

**Affiliations:** 1 Department of Periodontology, Dow International Dental College, Dow University of Health Sciences, Karachi, Pakistan; 2 Department of Operative Dentistry, Dr Ishrat-ul-Ebad Khan Institute of Oral Health Sciences, Dow University of Health Sciences, Karachi, Pakistan; 3 Department of Oral Biology, Dow Dental College, Dow University of Health Sciences, Karachi Sindh, 74200 Pakistan; 4 Department of Periodontology, Baqai Dental College, Baqai Medical University, Karachi, Pakistan; 5 Department of Operative Dentistry, Dr Ishrat-ul-Ebad Khan Institute of Oral Health Sciences, Dow University of Health Sciences, Karachi, Pakistan; 6 Department of Community Dentistry, Dow Dental College, Dow University of Health Sciences, Karachi, Pakistan; 7 Department of Oral Medicine, Baqai Dental College, Baqai Medical University, Karachi, Pakistan

**Keywords:** Dentin hypersensitivity, Gingival recession, Bonding agents

## Abstract

**Background:**

Dentin hypersensitivity (DH) is one of the most challenging and persistent dental complaints characterized by transient, intense pain triggered by various stimuli. It affects a significant portion of the global population, predominantly those aged 20–40. This study aims to evaluate the desensitizing efficacy of seventh-generation dentin bonding agents (Single Bond Universal by 3 M ESPE and Xeno-V + by Dentsply) against a control group using Bifluorid 12 by Voco in mitigating DH within a month of the follow-up period.

**Methods:**

This was a single-center, parallel-group, double-blind, controlled randomized clinical trial conducted at Dow University of Health Sciences, Karachi, Pakistan. A total of 105 patients with DH were allocated into three groups for this study. The patients were divided into three groups (Single Bond Universal by 3 M ESPE and Xeno-V + by Dentsply) and the control group containing fluoride varnish (Bifluorid 12 by Voco). Discomfort Interval Scale scores and Schiff Cold Air Sensitivity Scale scores were recorded at baseline, immediately after the intervention, after 01 weeks, and after 01 month.

**Results:**

All the materials demonstrated a statistically significant reduction in discomfort and sensitivity (DIS scores p-value 0.01) immediately after 01 week and over a period of 01 month after treatment compared with the baseline scores before application, with no single material proving superior over the one-month observation period. The study also provided insights into dental hygiene practices, with a significant majority using a toothbrush and sensitivity patterns, with cold stimuli being the most common cause of sensitivity.

**Conclusion:**

The study demonstrates that Single Bond Universal, Xeno V+, and Bifluorid 12 are equally effective in reducing dentin hypersensitivity, with no distinct superiority observed over a one-month period. The findings highlight the potential of fluoride varnishes as a less technique-sensitive and cost-effective option for treating DH, offering valuable insights for future research and clinical practice.

**Trial registration:**

NCT04225247 (https://clinicaltrials.gov/study/NCT04225247), Date of Registration: 13/01/2020. (Retrospectively registered).

## Introduction

Dentin hypersensitivity (DH) presents a significant clinical challenge for both patients and dental clinicians. This distressing condition is characterized by transient, intense pain, often triggered by stimuli such as cold, heat, or mechanical forces. DH affects a diverse global population, with a substantial prevalence of 1.34-98% [1]. Notably, individuals aged 20–40 years are particularly susceptible to this condition [1]. The root cause of DH lies in the exposure of dentinal tubules—microscopic channels that connect the pulp chamber to the tooth surface [2]. These tubules serve as vital conduits, allowing communication between the external environment and the dental pulp [3]. When factors like enamel wear, gum recession, or toothbrush abrasion lead to tubule exposure, nerve fibers near the odontoblasts within these tubules become activated, resulting in pain perception tubules [4].

Addressing DH involves a multifaceted approach including desensitizing toothpaste which is widely used and minimally invasive, desensitizing toothpaste containing compounds like potassium nitrate or strontium chloride provides relief [5]. Patients can continue managing DH using desensitizing toothpaste and maintaining optimal oral hygiene [6]. However, in-office treatments are necessary for severe cases, where professional interventions aim to occlude open dentinal tubules [7, 8]. Techniques include laser therapy, bioactive glasses containing Novamin, and dentin bonding agents [5, 9, 10]. Understanding the effectiveness of these bonding agents is crucial for enhancing patient comfort and overall quality of life [11]. DH management requires a tailored approach, balancing reversible treatments with irreversible interventions [12]. Continued research and advancements will refine our understanding and improve patient outcomes.

Seventh-generation bonding agents, such as Single Bond Universal and Xeno-V+, have gained prominence for their potential to alleviate sensitivity by sealing dentinal tubules and reducing nerve activation [13].

This clinical trial aims to determine the most effective treatment for DH, comparing seventh-generation bonding agents with fluoride varnish. The trial focuses on the bonding agents’ desensitizing efficiency over a month and examines the relationship between dental hygiene and sensitivity. It measures pain using discomfort scales at various stages post-treatment. This study aimed to identify the most effective desensitizing agent among the seventh-generation bonding agents for DH, addressing a key gap in understanding long-term treatment efficacy, by comparing their effectiveness with that of a control group treated with Bifluorid 12.

## Materials and methods

### Study design and setting

This was a double-blind, controlled randomized clinical trial conducted at Dr. Ishrat-ul-Ebad Khan Institute of Oral Health Sciences, Karachi, Pakistan, from March to October 2018. The study protocol was registered at NCT04225247 (https://clinicaltrials.gov/study/NCT04225247), (Study Identifier: NCT04225247) Date of Registration: 13/01/2020. (Retrospectively registered).

### Ethical approval

This study was approved by DUHS’s institutional review board, this non-invasive study ensured patient safety. Voluntary participation with signed consent forms-maintained confidentiality. Participants could withdraw at will, and no adverse reactions were reported.

#### Inclusion criteria

Participants, aged 18–45, were selected based on specific criteria ensuring relevance. Both genders with teeth hypersensitivity to air and tactile stimuli were considered, along with Miller Class I and II gingival recession. Oral hygiene, assessed by the Loe and Silness Plaque index (score of 1), no systemic illness, and voluntary consent were also vital for inclusion.

#### Exclusion criteria

Exclusions encompassed participants with carious teeth, fractured restorations, or cracked teeth, undergoing orthodontic or periodontal treatment, or recent dental bleaching. Those using painkillers, smokers, pregnant, or nursing females were also excluded.

#### Sample size

A total of 105 patients were allocated into three groups for this study. Sample size calculations were performed using PASS V.11 software. With an 80% power and a 95% confidence interval, the difference in the Discomfort Interval Scale score for hypersensitivity was determined to be 0.813, and the standard deviation was 1.39. Based on these parameters, the required sample size was estimated to be 26 patients per group. To account for potential dropouts, this number was conservatively increased to 35 patients per group, anticipating a dropout of 9 patients in each group. The allocation ratio of participants was 1:1:1.

#### Procedural details: clinical protocols and examination

Utilizing a consecutive sampling technique, participants meeting the inclusion criteria were selected. The diagnosis of dentin hypersensitivity was established through a thorough review of medical and dental histories, complemented by a clinical examination. The dentine hypersensitivity scores using DIS and Schiff scales were recorded at baseline.

As a standard protocol, each participant underwent non-surgical scaling and root debridement administered by the clinical examiner. The rationale behind this scaling was to effectively remove plaque and food debris, thereby ensuring the bonding agent and fluoride varnish could adhere optimally to a pristine tooth surface.

Subsequently, participants were randomly allocated into one of three groups. This allocation was executed by an independent individual using a computer-generated randomization method, enhancing the comparability and assessment of results. The groups were delineated based on the product names:

Group-I: Single Bond Universal by 3 M ESPE.

Group-II: Xeno V + by Dentsply.

Group-III: Bifluorid 12 by Voco (serving as the control group with fluoride varnish).

**Fig. 1:**
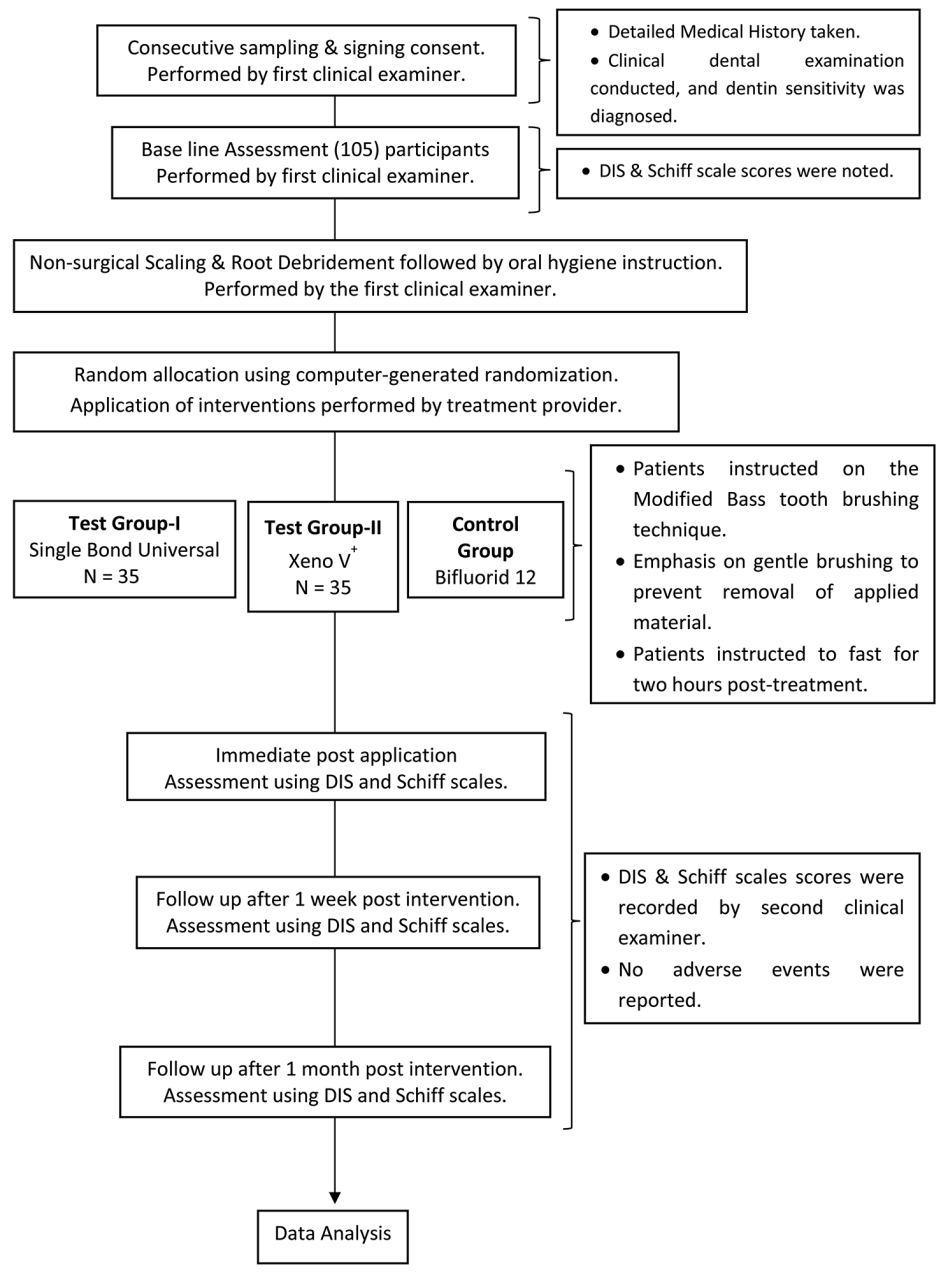
Study flow chart

For this study, we juxtaposed two “seventh-generation bonding agents” against a fluoride varnish control group, as shown in Fig. 1.

The study, designed as a double-blind, randomized clinical trial, had several layers of scrupulous planning in place to ensure the utmost validity of the results. In this context, neither the participant nor the clinical examiner and treatment provider knew which intervention was being administered. This ensured that any placebo effect or inadvertent treatment biases were minimized, if not eliminated.

The blinding process began when participants were randomly allocated into one of the three study groups. An independent individual, distant from the core research team, used a computer-generated randomization method to assign participants to the respective groups. These groups were only identifiable by product names: Group-I, Group-II, and Group-III. The containers or packaging for these products were likely made uniformly to look identical so as not to give any visual cues about their content.

As participants entered the examination room, they were unaware of the specific product they were to receive. The treatment provider was also unaware of the product’s identity. The interventions, whether Single Bond Universal, Xeno V+, or Bifluorid 12, were meticulously applied to the hypersensitive teeth of the participants.

The Discomfort Interval Scale (DIS) is an essential instrument for assessing the intensity of discomfort experienced by patients with dentin hypersensitivity, providing a subjective yet reliable metric for recording patient-reported outcomes [14]. Utilizing the Schiff sensitivity score alongside the DIS allows for a comprehensive evaluation of both the patient’s perception of pain and the clinical responsiveness of the dentin to external stimuli, which is pivotal in the assessment and management of dentin hypersensitivity [15].

Post-application, a second examiner blinded to the interventions documented the subjective responses immediately after application, one week after application, and after one month. Participants were scored on the Discomfort Interval Scales and the Schiff Cold Air Sensitivity Scale. This layer of blinding was crucial. By not knowing which treatment the participant received, this provider recorded data without any inherent biases or preconceived notions about the expected outcomes.

In instances where participants experienced severe or intolerable pain, necessitating the use of local anesthesia, the responses were taken the next day. This slight deviation from the immediate post-application recording was done to ensure that anesthesia didn’t skew the results, thereby preserving the blinding process’s integrity.

Before the application of the interventions, participants were given comprehensive oral hygiene instructions. They were particularly educated on the Modified Bass tooth brushing technique, which emphasizes the potential removal of applied materials through vigorous brushing. Specifically, participants were advised to position the bristles at a 45˚ angle along the gingival margin and to brush gently in vibrating, back-and-forth motions without disengaging the bristle tips. They were further guided to brush over the tooth crown, moving toward the occlusal surface. This regimen was recommended for all tooth surfaces, and brushing the tongue was also advocated.

The designated interventions were applied to the hypersensitive teeth during the same visit. The application was conducted under complete isolation, facilitated by cotton rolls, and overseen by the designated treatment provider.

In this clinical trial, the instructions from the manufacturers were followed for the application of Single Bond Universal by 3 M ESPE, Xeno V + by Dentsply, and Bifluorid 12 by Voco. Each desensitizing material was applied after removing plaque, cleaning, and drying the tooth surface. For Single Bond Universal and Xeno V+, the respective bottles were shaken, the bonding agent or adhesive was applied and scrubbed in for 20 s, then dried with an air syringe for 5 s, and finally, light-cured for 10 s. Bifluorid 12 involved shaking the bottle, applying a thin layer of the varnish, allowing it to soak for 10 to 20 s, and drying with an air syringe if necessary. These procedures were meticulously carried out during the trial to ensure the integrity of the application process according to the manufacturers’ guidelines.

After the application, scores on the Discomfort Interval Scale and Schiff Cold Air Sensitivity Scale were diligently noted. These scores were recorded at the baseline, immediately post-intervention, after one week, and after one month. The Discomfort Interval Scale scores were assiduously recorded for every participant using a dental explorer, moving in both mesial and distal directions on the exposed tooth surface for tactile stimulation.

For the Schiff Cold Air Sensitivity Scale, an air syringe system was deployed to gauge the evaporative response on participants’ affected teeth, with scores being duly documented. The treatment provider applied dentin bonding agents and fluoride varnish.

Post-application, participants were uniformly advised to abstain from eating or drinking for two hours. All records, capturing the subjective responses of the participants, were scrupulously documented by the clinical examiner.

Grade 4 (DIS score) and Grade 3 (Schiff Cold Air Sensitivity Scale score) application of dentin bonding agents and fluoride varnish were done under local anesthesia, and responses were taken the next day.

**a) Discomfort Interval Scale (DIS)**[16]

- 0 = No pain.
- 1 = Mild pain.
- 2 = Moderate pain.
- 3 = Severe pain.
- 4 = Intolerable pain.

**b) Schiff Cold Air Sensitivity Scale**[17].

- 0 = Subject does not respond to air stimulus.
- 1 = Subject responds to air stimulus but does not request discontinuation of stimulus.
- 2 = Subject responds to air stimulus and requests discontinuation or moves from stimulus.
- 3 = Subject responds to air stimulus, considers stimulus to be painful and requests discontinuation of stimulus.

## Calibrations

Prior to the commencement of the main study, a rigorous examiner calibration process was conducted to ensure consistency and reliability in the application of the Discomfort Interval Scale (DIS) and the Schiff Cold Air Sensitivity Scale. The Department of Periodontology at Dow University of Health Sciences facilitated this training and calibration over five days, involving 20 participants distinct from those in the main study.

Clinical examiners 1 and 2, who were designated for the study, participated in the calibration process. They independently assessed the pain responses of the participants using both the DIS and Schiff scale. To establish intra-examiner reliability, each examiner reassessed the same participants after a suitable interval without referencing their initial scores, ensuring a blind re-evaluation.

The calibration results demonstrated high intra-examiner reliability for both examiners on the DIS, with examiner 1 achieving a reliability score of 0.88 and examiner 2 achieving 0.90. Similarly, for the Schiff scale, Examiner 1, and Examiner 2 reported reliability scores of 0.82 and 0.88, respectively, indicating strong consistency in their assessments.

Inter-examiner reliability was also calculated to determine the degree of agreement between the two examiners. For the DIS, the inter-examiner reliability was 0.75, while the Schiff scale was 0.80. These results suggest a substantial level of agreement and confirm the effectiveness of the calibration process.

Through this comprehensive calibration, we ensured that the examiners were well-aligned in their understanding and application of the scales, thus bolstering the validity of the subsequent measurements in our study.

## Statistical analysis

The collected data were processed and analyzed using SPSS software (version 22). For categorical variables, such as brushing habits, brushing frequency, gender, frequency of sensitivity, and types of sensitivity, frequencies and percentages were calculated. The mean and standard deviation were determined by age.

Cross-tabulation offered in-depth insight into the baseline discomfort interval scale scores across the three groups before applying the desensitizing material. This approach also facilitated a better understanding of the distribution of Schiff scores among the groups in response to various stimuli.

The normality of the DIS and Schiff scores was assessed using both the Kolmogorov-Smirnov Test and the Shapiro-Wilk Test. For every time interval (DIS_before, DIS_immediately, DIS_1wk, and DIS_1mnth), the tests yielded significance values (p-values) less than 0.05 (specifically, 0.000), indicating a significant deviation from a normal distribution. Similarly, for the Schiff scores at all time intervals (Schiff_before, Schiff_immediate, Schiff_1wk, and Schiff_1month), the p-values were consistently less than 0.05, signaling a marked departure from a normal distribution.

To discern differences in both the DIS and Schiff scores across the three desensitizing materials at varied time intervals, the Kruskal-Walli’s test was deployed. Furthermore, to evaluate the changes in the Discomfort Interval Scale (DIS) and Schiff scores pre- and post-application of each desensitizing material (Single Bond Universal, Xeno V+, and Bifluorid 12) at the designated time points (before, immediately, one-week post-application, and one-month post-application), the Wilcoxon Signed-Rank Test was utilized. The threshold for statistical significance was established at less than 0.05.

## Results

The average age was 27.75 years, and the standard deviation was 7.43. A vast majority (96.2%) of the participants used a toothbrush for oral hygiene, with only a minor proportion (3.8%) using a Miswak as shown in Table 1.

Over half of the participants (52.4%) brush their teeth once daily. A significant portion (44.8%) brush their teeth twice daily, while a small percentage (2.8%) brush only once a week. Sensitivity was reported by participants at varying degrees. While (9.5%) always experience sensitivity, (21.9%) feel it most of the time—the largest segment, (41%) report experiencing sensitivity sometimes, and (27.6%) only occasionally.

Cold stimuli were most common, reported by (51.4%) of participants, followed by hot and cold sensitivities at (24.8%). Sweet sensitivity was noted by only (1.9%) of respondents. Some experienced multiple sensitivities: cold and sweet (10.4%), cold and air (5.6%), and hot, cold, and sweet (4.8%). Female participants comprised (65.7%), males (34.3%) of the study group.

**Table 1:** Demographic overview of dental hygiene practices and tooth sensitivity patterns among participants

Brushing Habits	Frequency (%)
Miswak	04 (3.8%)
Brush	101 (96.2%)
**Brushing Frequency**	
Once a Week	03 (2.8%)
Once Daily	55 (52.4)
Twice Daily	47 (44.8%)
**Frequency of Sensitivity**	
Always	10 (9.5%)
Most of the Time	23 (21.9%)
Sometime	43 (41%)
Occasionally	29 (27.6%)
**Sensitivity**	
Hot	1 (1%)
Cold	54 (51.4%)
Sweet	02 (1.9%)
Cold + Sweet	11 (10.4%)
Cold + Air	06 (5.6%)
Hot + Cold	26 (24.8%)
Hot + Cold + Sweet	05 (4.8%)
**Gender**	
Male	36 (34.3%)
Female	69 (65.7%)

A cross-tabulation was conducted to explore the baseline DIS scores across three groups before applying desensitizing material, as shown in Table 2.

In the DIS scores for Single Bond Universal (Group 1), mild discomfort was reported by 10 participants (9.5%), moderate by 17 (16.2%), severe by 5 (4.8%), and intolerable by 3 (2.9%). For Xeno V+ (Group 2), mild discomfort was reported by 11 participants (10.5%), moderate by 15 (14.3%), severe by 7 (6.7%), and intolerable by 2 (1.9%). Bifluorid 12 (Group 3) had 11 participants (10.5%) with mild discomfort, 18 (17.1%) with moderate, 5 (4.8%) with severe, and 1 (1.0%) with intolerable discomfort.

For Group 1 Schiff scores, 7 participants (6.7%) responded to the air stimulus but did not request discontinuation, 25 (23.8%) responded and requested discontinuation, and 3 (2.9%) found it painful and requested discontinuation. In Group 2, 9 participants (8.6%) responded without discontinuation, 21 (20%) requested discontinuation, and 5 (4.8%) found it painful and requested discontinuation. In Group 3, 6 participants (5.7%) did not request discontinuation, 23 (21.9%) requested discontinuation, and 6 (5.7%) found it painful and requested discontinuation.

**Table 2:** Baseline DIS scores for all three groups before the application of desensitizing material

Discomfort Interval Scale (DIS)	Single Bond Universal Group 1 *N*(%)	Xeno V+ Group 2 *N*(%)	Bifluorid 12 Group 3 *N*(%)	Total *N*(%)
Mild	10(9.5)	11(10.5)	11(10.5)	32(30.5)
Moderate	17(16.2)	15(14.3)	18(17.1)	50(47.6)
Severe	5(4.8)	7(6.7)	5(4.8)	17(16.2)
Intolerable	3(2.9)	2(1.9)	1(1.0)	6(5.7)
Total	35(33.3)	35(33.3)	35(33.3)	105(100)
**Schiff Cold Air Scale**	**Group 1** **N(%)**	**Group 2** **N(%)**	**Group 3** **N(%)**	**Total** **N(%)**
Subject responds to air stimulus but does not request discontinuation	7(6.7)	9(8.6)	6(5.7)	22(21)
Subject responds to air stimulus and requests discontinuation	25(23.8)	21(20)	23(21.9)	69(65.7)
Subject considers the stimulus to be painful and requests discontinuation	3(2.9)	5(4.8)	6(5.7)	14(13.3)
Total	35(33.3)	35(33.3)	35(33.3)	105(100)
Cross tabulations				

Utilizing the Kruskal-Walli’s test, we investigated differences in both DIS and Schiff scores among three distinct materials: Single Bond Universal, Xeno V+, and Bifluorid 12, as shown in Table 3. These differences were measured at three separate time intervals: immediately, after one week, and after one month.

For the DIS_immediately measurement, both Single Bond Universal and Bifluorid 12 exhibited a mean rank of 53.50 across their 35 samples. In contrast, Xeno V + registered a slightly lower mean rank of 52.00, also based on 35 samples.

During the DIS_1wk measurement, Single Bond Universal presented a mean rank of 52.56 derived from 34 samples. Xeno V + followed with a mean rank of 53.96 based on 35 samples. Notably, Bifluorid 12 showcased the most modest mean rank of 50.99, also accumulated from 35 samples.

In the DIS_1mnth assessment, Single Bond Universal recorded a mean rank of 51.09 based on 33 samples. Xeno V + indicated a slightly higher mean rank of 52.37, evaluated from 35 samples. Meanwhile, Bifluorid 12 achieved a mean rank of 51.00, drawn from its 34 samples.

Based on the Schiff scores, the immediate measurements interestingly showed all three materials, Single Bond Universal, Xeno V+, and Bifluorid 12, having an identical mean rank of 53.00 based on 35 samples.

For the Schiff_1wk measurement, Single Bond Universal posted a mean rank of 50.53 from 34 samples, whereas both Xeno V + and Bifluorid 12 shared the same mean rank of 53.46 based on 35 samples.

In the Schiff_1month assessment, Single Bond Universal had a mean rank of 50.05 from 33 samples, Xeno V + registered a mean rank of 52.87 from 35 samples, and Bifluorid 12 reported a mean rank of 51.50 from 34 samples. In summation, neither the DIS nor Schiff scores displayed any significant difference in the three materials over the examined time intervals, as shown in Table 3.

**Table 3:** Differences in DIS and Schiff scores among three materials and time points

Measurement Time	Material	*N*	Mean Rank	Chi-Square	df	*p*-value
DIS_immediately	Single Bond Universal	35	53.50	1.010	2	0.604
	Xeno V+	35	52.00			
	Bifluorid 12	35	53.50			
DIS_1wk	Single Bond Universal	35	52.56	1.042	2	0.594
	Xeno V+	35	53.96			
	Bifluorid 12	35	50.99			
DIS_1mnth	Single Bond Universal	33	51.09	0.242	2	0.886
	Xeno V+	35	52.37			
	Bifluorid 12	35	51.00			
Schiff_immediately	Single Bond Universal	35	53.00	0.000	2	1.000
	Xeno V+	35	53.00			
	Bifluorid 12	35	53.00			
Schiff _1wk	Single Bond Universal	35	50.53	1.144	2	0.564
	Xeno V+	35	53.46			
	Bifluorid 12	35	53.46			
Schiff _1mnth	Single Bond Universal	35	50.05	0.933	2	0.627
	Xeno V+	35	52.87			
	Bifluorid 12	35	51.50			

Kruskal-Walli’s test, Schiff Cold Air Sensitivity Scale, Discomfort Interval Scale

### For the discomfort interval scale (DIS)

Immediately post-application of the Single Bond Universal, the mean rank was 17.50 (sum of ranks 595.00), yielding a Z-statistic of -5.173 and a p-value < 0.0001, indicating significant deviation from the baseline. At one week, the mean rank was 17.00 (sum of ranks 561.00), with a Z-statistic of -5.102 and p-value < 0.0001, maintaining significance. After one month, the mean rank was 16.50 (sum of ranks 528.00), yielding a Z-statistic of -5.029 and p-value < 0.0001, showing sustained significance.

#### For the schiff scores

Immediately post-application, the mean rank was 17.50 (sum of ranks 595.00), yielding a Z-statistic of -5.321 and p-value < 0.0001, signifying significant deviation from baseline. At one week, the mean rank was 16.50 (sum of ranks 528.00), with a Z-statistic of -5.208 and p-value < 0.0001, maintaining significance. After one month, the mean rank was 16.00 (sum of ranks 496.00), yielding a Z-statistic of -5.118 and p-value < 0.0001, indicating sustained significance. In conclusion, Single Bond Universal application led to significant changes in DIS and Schiff scores at all time points compared to baseline (Table 4), with consistently low p-values (< 0.0001) highlighting its significant impact.

**Table 4:** DIS and Schiff scores before and after the application of a single bond universal

Comparison	Mean Rank		Sum of Ranks	Z-statistic			*p*-value
DIS_immediately vs. DIS_before	17.50		595.00	-5.173			< 0.0001
DIS_1wk vs. DIS_before	17.00		561.00	-5.102			< 0.0001
DIS_1mnth vs. DIS_before	16.50		528.00	-5.029			< 0.0001
Schiff_immediate vs. Schiff_before	17.50		595.00	-5.321			< 0.0001
Schiff_1wk vs. Schiff_before	16.50		528.00	-5.208			< 0.0001
Schiff_1month vs. Schiff_before	16.00		496.00	-5.118			< 0.0001
DIS and Schiff scores before and after the application of a Xeno V+							
**Comparison**	**Mean Rank**	**Sum of Ranks**				**Z-statistic**	**p-value**
DIS_immediately vs. DIS_before	18.00	630.00				-5.233	< 0.0001
DIS_1wk vs. DIS_before	18.00	630.00				-5.236	< 0.0001
DIS_1mnth vs. DIS_before	18.00	630.00				-5.247	< 0.0001
Schiff_immediate vs. Schiff_before	17.50	595.00				-5.246	< 0.0001
Schiff_1wk vs. Schiff_before	17.50	595.00				-5.249	< 0.0001
Schiff_1month vs. Schiff_before	17.50	595.00				-5.249	< 0.0001
DIS and Schiff scores before and after the application of a Bifluorid 12							
**Comparison**	**Mean Rank**	**Sum of Ranks**			**Z-statistic**		**p-value**
DIS_immediately vs. DIS_before	34	17.50			595.00		-5.173
DIS_1wk vs. DIS_before	33	17.00			561.00		-5.102
DIS_1mnth vs. DIS_before	32	16.50			528.00		-5.029
Schiff_immediate vs. Schiff_before	34	17.50			595.00		-5.321
Schiff_1wk vs. Schiff_before	32	16.50			528.00		-5.208
Schiff_1month vs. Schiff_before	31	16.00			496.00		-5.118

Wilcoxon signed rank test, Schiff Cold Air Sensitivity Scale, Discomfort Interval Scale

Table 4 offers a statistical evaluation of the DIS and Schiff scores before and after the application of Xeno V + at various intervals.

#### For the discomfort interval scale (DIS)

Immediately after applying Xeno V+, the mean rank was 18.00 (sum of ranks 630.00), yielding a Z-statistic of -5.233 and p-value < 0.0001, indicating significant deviation from baseline. One week later, the mean rank remained 18.00 (sum of ranks 630.00), with a Z-statistic of -5.236 and p-value < 0.0001, maintaining significance. After one month, the mean rank persisted at 18.00 (sum of ranks 630.00), with a Z-statistic of -5.247 and p-value < 0.0001, highlighting a notable difference from baseline.

#### For schiff scores

Right after applying Xeno V+, the mean rank was 17.50 (sum of ranks 595.00), with a Z-statistic of -5.246 and p-value < 0.0001, indicating a significant alteration from baseline. One week later, the mean rank remained at 17.50 (sum of ranks 595.00), with a Z-statistic of -5.249 and p-value < 0.0001, sustaining significant difference. After one month, the mean rank persisted at 17.50 (sum of ranks 595.00), with a Z-statistic of -5.249 and p-value < 0.0001, confirming continued distinction from baseline.

In conclusion, Xeno V + application resulted in marked statistical differences in DIS and Schiff scores at all timeframes (immediately, 1 week, and 1 month) compared to baseline (Table 4), with consistent p-values (< 0.0001) emphasizing its profound influence.

#### For the discomfort interval scale (DIS)

Immediately after applying Bifluorid 12, the mean rank was 34 (sum of ranks 595.00), with a Z-statistic of -5.173, indicating significant difference from baseline. One week later, the mean rank decreased to 33 (sum of ranks 561.00), yielding a Z-statistic of -5.102, maintaining significance. After one month, the mean rank further reduced to 32 (sum of ranks 528.00), with a Z-statistic of -5.029, confirming sustained significance.

#### For the schiff scores

Immediately post-application of Bifluorid 12, the mean rank was 34 (sum of ranks 595.00), with a Z-statistic of -5.321, indicating significant deviation from baseline. One week later, the mean rank decreased to 32 (sum of ranks 528.00), yielding a Z-statistic of -5.208, maintaining significance. After one month, the mean rank further reduced to 31 (sum of ranks 496.00), with a Z-statistic of -5.118, confirming sustained significance. In conclusion, Bifluorid 12 induced statistically significant changes in DIS and Schiff scores at all observed durations (immediately, 1 week, and 1 month) compared to respective baselines (Table 4), with negative Z-statistics underscoring its substantial impact.

## Summary of results

In summary, all three desensitizing materials (Single Bond Universal, Xeno V+, and Bifluorid 12) significantly reduced discomfort and sensitivity compared to baseline, indicating their effectiveness in managing dental sensitivity. No material showed superiority over others at one month, suggesting their comparable benefits. Thus, the use of these materials can be deemed beneficial for addressing dental sensitivity, irrespective of the specific product chosen.

## Discussion

The present study provides insightful findings on dental hygiene practices, tooth sensitivity patterns, and the efficacy of different desensitizing materials over time. The present study tested the efficacy of seventh-generation dentin bonding agents as desensitizers, taking fluoride varnish as the control group in patients with dentin hypersensitivity.

The mean age reported in the present study was 27.75 ± 7.43, indicating a relatively young adult cohort. This demographic characteristic is consistent with the target population of many oral health studies focused on individuals aged 18–35 years to assess oral health behaviors [18–20]. Graf et al., in their study, stated that sensitivity is commonly seen in people 20 to 40 years of age [21, 22]. Many studies described different prevalent age groups of dentin hypersensitivity; Vijaya reported 18–27 years [23], Amarasena recorded 30–49 years [24], Bahsi documented 40–49 years [25], and Rees J published 40–50 years [26].

The gender distribution, with more female participants, aligns with research suggesting that women are more likely to report tooth sensitivity, potentially due to differences in perception or reporting behaviors [1]. In the present study, most patients were female, constituting 65.7%, suggesting DH is more commonly observed in females. Feng and companions reported a higher prevalence of DH in females, corresponding to the results of the present study [27]. In Northwest America, a study documented a higher frequency of DH in females, corroborating this study [28]. A study conducted in Australia also found that DH most commonly affects females [24]. Bahsi et al., working on the prevalence of DH [25] and another study on cervical dentin hypersensitivity [29], recorded DH more frequently in females, just like the present study. However, few studies documented a higher prevalence of dentin hypersensitivity among males, contrary to the present study’s findings [30].

Regarding dental hygiene, 96.2% of participants used a toothbrush, while 3.8% used Miswak. This aligns with global oral hygiene practices, where using a toothbrush is predominant. However, the use of Miswak is culturally specific. It has been reported to have similar efficacy to toothbrushing in plaque reduction, as noted in the study by Al-Otaibi et al. (2003) [31].

The frequency of brushing habits revealed that 52.4% of participants brushed once and 44.8% twice daily. It is slightly less than what dental associations recommend globally, which recommends brushing twice a day [32]. It is worth comparing these findings with those of Folayan et al. (2020), who reported a higher prevalence of twice-daily brushing in a similar age group [33]. This variance in dental hygiene habits could be influenced by cultural, educational, or economic factors.

This study reported that cold stimuli were the most common cause of sensitivity. In the present study, almost 51% of patients were sensitive to cold stimuli; the result parallels the study conducted in Karachi in 2016, reporting 71% sensitivity to cold [20]. This observation is corroborated by other studies, such as Rees and Addy (2002), who found that cold stimuli are the most frequently reported trigger for dentinal hypersensitivity [34, 35].

In the present study, lower incisors were the most commonly affected teeth with dentin hypersensitivity, followed by upper incisors. Gingival recession is one of the reasons for the frequent involvement of lower incisors. Teeth involved in hypersensitivity showed much variability in different parts of the world. A study on the prevalence of dentin hypersensitivity in Hong Kong reported lower incisors as the most sensitive teeth [26]. Another study observing the frequency of DH in Pakistan documented mandibular incisors as the most commonly involved teeth in hypersensitivity [20]. Frequent involvement of lower incisors was also reported in Indian Rural Punjab [35]. The results of all these studies are in agreement with the findings of the present study.

In contrast to the results of the present study, a pilot study on CDS reported hypersensitivity in molars. A cross-sectional study on buccal cervical sensitivity in the United Kingdom reported molars and premolars as the most commonly affected teeth, and they also reported lower central incisors to be the least sensitive teeth [36]. Multiple researches also reported sensitivity in bicuspids [5, 36].

All the materials (Single Bond Universal by 3 M ESPE, Xeno-V + by Dentsply, and Bifluorid 12 by Voco) used in the present randomized clinical trial significantly reduced dentin hypersensitivity immediately after application of the desensitizing material after 01 week and over 01 month, proving seventh generation dentin bonding agents as a successful treatment option for treating dentin hypersensitivity.

Bifluorid 12, a fluoride varnish, contains calcium and sodium fluoride. It acts by forming precipitates of Calcium Fluoride within orifices of tubules and on the tooth surface, occluding the patent tubules by crystallizing Sodium Fluoride [37]. TM Silva et al. evaluated the efficacy of desensitizing agents for the relief of dentin hypersensitivity using a split-mouth model; they declared a significant reduction in hypersensitivity after application of Bifluorid 12, for tactile and evaporative stimulus for a period of three and four weeks, respectively [38], corresponding to the results of the present study. An in vivo study conducted in 2010 tested the efficacy of Bifluorid 12 and found it useful for thermal dentin hypersensitivity for 01 month, just like the present study [39], but could not maintain it for mechanical dentin hypersensitivity for 01 month.

Another randomized controlled trial comparing different desensitizing agents reported a significant reduction in hypersensitivity immediately post-treatment and 02 months post-treatment using fluoride varnish and potassium salt solution, congruent with our findings [40]. Nardi et al. also declared a significant reduction in sensitivity after 30 and 90 days of application of 02 different varnishes [41].

Loveren et al., in their study, indicated the use of DBA as an in-office professional treatment option for treating dentin hypersensitivity [42]. A 03 months follow-up study found no difference while checking the effect of one bottle adhesives in treating hypersensitivity with or without acid etching [43]. In a comparative study of different generations of DBA, the fifth generation was declared more efficient in reducing DH [44]. They also reported that the efficiency of dentin bonding agents to reduce hypersensitivity decreases with time. The study on tooth cervical hypersensitivity tested the effectiveness of the desensitizing ability of glutaraldehyde-based HEMA and one bottle bonding agent and found no differences after 08 weeks follow-up. However, after 09 months, Gluma showed less hypersensitivity and was reported to be more durable [45]. While comparing the effectiveness of one bottle of self-etching adhesive, dentin desensitizer and a combination of both for treating dentin hypersensitivity, Shruti et al. reported significant reduction in all 03 groups for 06 weeks. However, dentin desensitizer and a combination of adhesive with desensitizer showed greater reduction than one bottle of self-etching adhesive [46].

A clinical trial evaluated the efficacy of self-etch adhesives on hypersensitivity after periodontal surgery and found its use beneficial [47]. A randomized clinical trial evaluating dentin desensitizers reported that all the materials used in the study significantly reduced hypersensitivity immediately, and adhesives relieved the pain over 01 month similar to the results of this study [39].

Discrepancies in the findings of various studies could be attributed to differences in the study populations, methodologies, and local practices or perceptions. The contrasting results regarding the most affected teeth and long-term efficacy of treatments underline the complexity of dental health studies and the influence of diverse confounding factors on their outcomes.

In the current study, different desensitizing materials showed a significant reduction in hypersensitivity, with no single material superior over one month. These results are similar to a systematic review where bonding agents have resulted in the immediate term and midterm reduction (one-month duration) in DH [38]. Bonding agents in this systematic review have shown efficacy similar to stannous fluoride, glutaraldehyde with HEMA, hydroxyapatite, glass ionomer cement and Laser groups in midterm reduction of DH [48]. A recent double-blind study compared the efficacy of Colgate Pro-Relief In-office, Admira Protect (Voco), and Bifluorid 12 (Voco) in a cohort of 40 patients diagnosed with DH. The study revealed that Bifluorid 12 demonstrated a more sustained desensitizing impact than Colgate Pro-Relief (applied in the office setting) in response to tactile and evaporative stimuli [38].

Our study demonstrated that seventh-generation dentin bonding agents and Bifluorid 12 were equally effective at reducing DH. No statistical difference was found in terms of their effectiveness and superiority over one another, as shown in Table 3. The results of this study hold important clinical implications, given that seventh-generation bonding agents, which consist of two components – etch and bond – are susceptible to varying degrees of solvent evaporation. Additionally, these agents are sensitive to technique and moisture, more time-intensive, and require multiple steps, making them costlier compared to the single-step application of Bifluorid 12.

This study’s design and execution incorporate several notable strengths, highlighting its contribution to the field of dentistry, particularly in the management of DH. A key feature of this research is its adoption of a double-blind methodology, a critical aspect in clinical trials aimed at reducing reporting bias. By ensuring that neither the participants nor the researchers were aware of the treatment allocation, the study significantly mitigates the potential for subjective influence on the outcomes, thereby enhancing the integrity and validity of the results.

Central to this study is its focus on evaluating less technique-sensitive desensitizing agents, specifically the seventh-generation dentin bonding agents. This choice reflects a thoughtful consideration of clinical practicality and efficacy. In treating DH, the ease and simplicity of the application process are crucial, especially in a clinical setting where time and resources are often limited. By providing empirical evidence supporting the use of these agents, the study makes a significant contribution to clinical practice, offering a viable and efficient alternative for treating DH.

Furthermore, the study distinguishes itself as a pioneering effort in its demographic context. It is the first in our population to compare seventh-generation dentin bonding agents and fluoride varnishes as desensitizers. This comparative approach not only broadens the understanding of the effectiveness of these treatments but also contextualizes their utility within a specific population, offering valuable insights for localized clinical practices.

The study innovatively employs subjective response measures to assess the desensitizing agents’ efficacy. While subjective responses can vary significantly among individuals, they provide crucial insights into the patient’s perspective and experience of relief, which is a central concern in clinical outcomes. This patient-centered approach to evaluating treatment efficacy reflects a holistic understanding of clinical success, transcending mere clinical measurements.

Recognizing the study’s constraints and opportunities for further inquiry, it should be noted that the research spanned a short follow-up period of just one month. This duration, while providing initial insights into the effectiveness of the treatments, opens avenues for subsequent studies with extended follow-up periods. Longer follow-up durations in future research could yield more comprehensive data, offering a deeper understanding of these treatments’ long-term efficacy and sustainability.

In conducting our research on the effectiveness of desensitizing materials for dental hypersensitivity, we recognized the potential influence of various confounding variables. These confounders were differences in participant characteristics (such as age, gender, and baseline oral health), individual variations in pain perception, adherence to oral hygiene practices, and environmental factors that could affect dental sensitivity.

To mitigate the impact of these confounding variables, our study design incorporated several methodological safeguards:

Our study’s foundation was a randomized controlled trial design. This strategy is pivotal in neutralizing the effects of both known and unknown confounders, thereby ensuring a fair comparison among the treatment modalities. The double-blind nature of our study played a critical role in minimizing biases. This approach was crucial in eliminating biases related to treatment administration and participant response, significantly reducing placebo effects and observer biases. We established stringent inclusion and exclusion criteria for creating a more uniform study population, reducing the impact of confounding variables related to individual dental health histories and systemic health conditions.

Before applying the desensitizing agents, all participants underwent a standardized treatment protocol, including non-surgical scaling and root debridement. Additionally, uniform oral hygiene instructions were provided to all participants. These standardized procedures ensured that each participant started with a similar baseline, thus reducing variability in initial oral health conditions. To ensure consistency in the application of outcome measures, our examiners underwent a thorough calibration process. This step was pivotal in minimizing inter-examiner variability, particularly in interpreting and applying the Discomfort Interval Scale and Schiff Cold Air Sensitivity Scale. The inclusion of a control group and the application of standardized measurement scales provided a structured framework for assessing treatment outcomes. This approach allowed for a more objective evaluation of the effectiveness of the desensitizing materials, reducing the influence of subjective biases.

## Recommendations

Future studies should aim to extend the duration of follow-up periods. This will allow for a more comprehensive evaluation of desensitizing materials’ long-term efficacy and sustainability. The study underscores the importance of understanding and documenting oral hygiene practices in relation to DH. Future research should continue to explore how different hygiene habits impact the onset and severity of DH and how they may interact with the effectiveness of desensitizing agents. An important finding is the equivalency noted between different desensitizing agents in the short term. Future research should aim to build on this by comparing a broader range of agents over extended periods to determine the most effective and practical solutions for DH. Given the subjective nature of DH analysis, the results from different clinical trials must be interpreted with caution. Researchers and practitioners should consider the contextual and individual variations that might affect the study outcomes and their applicability in different clinical settings.

By implementing these recommendations, future research can significantly contribute to the understanding and management of Dentin Hypersensitivity, ultimately leading to more effective and patient-centric treatment approaches.

## Conclusion

This study on DH found that all three desensitizing materials tested (Single Bond Universal, Xeno V+, and Bifluorid 12) effectively reduced sensitivity, without any one material outperforming the others over a one-month period. The study highlights the immediate and midterm benefits of these materials, including seventh-generation dentin bonding agents and fluoride varnishes. This study underlines the influence of dental hygiene practices and cold sensitivity on DH, advocating for personalized treatment approaches. This study emphasizes the importance of considering patients’ subjective experiences in evaluating treatment effectiveness and stresses the need for patient education. It suggests that different desensitizing agents have comparable short-term and mid-term effects and advises caution in interpreting clinical results due to DH’s subjective nature. Overall, the study contributes significantly to understanding DH by combining clinical efficacy with a focus on patient experiences and rigorous research design, suggesting seventh-generation dentin bonding agents as a promising treatment modality for DH.

## Data Availability

he datasets analysed during the current study are available from the corresponding author on reasonable request.
